# Exchangeable Ions Are Responsible for the *In Vitro* Antibacterial Properties of Natural Clay Mixtures

**DOI:** 10.1371/journal.pone.0064068

**Published:** 2013-05-17

**Authors:** Caitlin C. Otto, Shelley E. Haydel

**Affiliations:** 1 School of Life Sciences, Arizona State University, Tempe, Arizona, United States of America; 2 The Biodesign Institute Center for Infectious Diseases and Vaccinology, Arizona State University, Tempe, Arizona, United States of America; Rockefeller University, United States of America

## Abstract

We have identified a natural clay mixture that exhibits *in vitro* antibacterial activity against a broad spectrum of bacterial pathogens. We collected four samples from the same source and demonstrated through antibacterial susceptibility testing that these clay mixtures have markedly different antibacterial activity against *Escherichia coli* and methicillin-resistant *Staphylococcus aureus* (MRSA). Here, we used X-ray diffraction (XRD) and inductively coupled plasma – optical emission spectroscopy (ICP-OES) and – mass spectrometry (ICP-MS) to characterize the mineralogical and chemical features of the four clay mixture samples. XRD analyses of the clay mixtures revealed minor mineralogical differences between the four samples. However, ICP analyses demonstrated that the concentrations of many elements, Fe, Co, Cu, Ni, and Zn, in particular, vary greatly across the four clay mixture leachates. Supplementation of a non-antibacterial leachate containing lower concentrations of Fe, Co, Ni, Cu, and Zn to final ion concentrations and a pH equivalent to that of the antibacterial leachate generated antibacterial activity against *E. coli* and MRSA, confirming the role of these ions in the antibacterial clay mixture leachates. Speciation modeling revealed increased concentrations of soluble Cu^2+^ and Fe^2+^ in the antibacterial leachates, compared to the non-antibacterial leachates, suggesting these ionic species specifically are modulating the antibacterial activity of the leachates. Finally, linear regression analyses comparing the log_10_ reduction in bacterial viability to the concentration of individual ion species revealed positive correlations with Zn^2+^ and Cu^2+^ and antibacterial activity, a negative correlation with Fe^3+^, and no correlation with pH. Together, these analyses further indicate that the ion concentration of specific species (Fe^2+^, Cu^2+^, and Zn^2+^) are responsible for antibacterial activity and that killing activity is not solely attributed to pH.

## Introduction

Recent epidemiological studies have demonstrated a steady increase in infections due to antibiotic-resistant bacteria [1], [2]. These trends of increasing antibiotic resistance demonstrate an ongoing need to develop novel therapeutic treatments for bacterial infections. Clays have been used for medicinal applications throughout recorded history. The ancient tablets of Nippur, written approximately 5,000 years ago, listed clays as medicament for healing wounds and stopping “fluxes from the body”. The Ebers Papyrus, the world’s oldest medical text, dated approximately 1600 BC, lists clay as a mineral remedy for ailments such as diarrhea, dysentery, tapeworm, hookworm, wounds, and abscesses [3]. During the late 19th century, clays were used as topical treatments for surgical wounds with demonstrated beneficial effects on pain management, inflammation, putrefaction, and healing processes [4]. Reinbacher [5] describes German physician Dr. Julius Stumpf’s treatment strategy in 1898 of a patient who had long been suffering from a deep and suppurating ulcer of the tibia. This patient refused amputation, so the physician began treatment with a thick layer of fine clay powder. The wound immediately stopped producing a malodorous discharge and after four days of repeated clay application and bandaging, the ulcer healed. More recently, clays have been applied in a similar manner for the treatment of bacterial infections caused by *Mycobacterium ulcerans*, the causative agent of Buruli ulcer, which is a difficult-to-treat necrotic skin disease. A French humanitarian working in the Ivory Coast of Africa applied thick clay poultices daily, alternating between two types of clay, to individuals afflicted with Buruli ulcer. After several months of treatment, the infections often healed with some scarring and a resumption of normal motor function [6]–[8].

The healing property of clay has been attributed to both the physical and chemical properties of the minerals [8]–[10]. Clay minerals have a negative surface charge that allows the free exchange of particles from the environment such as bacteria, viruses, proteins, nucleic acids, and cations [11]. In particular, kaolinite, talc, and smectite clay minerals are highly absorptive and capable of adhering to the skin, thus offering mechanical protection against external physical or chemical agents and serving as dermatological protectors [11].

While clay minerals have been used with favorable outcomes for centuries in pharmaceutical applications, technology, and dermopharmacy as excipients and as active substances, their use in medical applications does not come without possible side effects. Several important factors that contribute to mineral toxicity include (i) site of ingress to the body (inhalation, ingestion, absorption), (ii) duration of exposure to the particles, and (iii) particle size [9]. The predominant pathogenicity of clay minerals is due to inhalation of the minerals, potentially leading to lung cancer, pneumoconiosis, or silicosis [12]. Moreover, due to their net negative charge, clays bind toxic metals to their surface. When in a hydrated environment, the ionic species adsorbed to clay surfaces can be exchanged into the surrounding medium in a manner that depends on the ionic strength of the aqueous medium and cation selectivity of the clay [13]. Mascolo *et al*. [14] fed mice one of three different clay samples and detected increased levels of As, Ni, and Se in urine samples, thus indicating that desorptive processes occur after clay ingestion. In addition, Tateo *et al*. [15] showed that ions, specifically Li, Sr, B, I, Rb, Br, Ba, Na, Cl, Se, and Ca, bound to pelotherapy clays were released from the mineral surface and penetrated human skin. While several of these elements are essential, caution must be taken to ensure that the transferred ion concentrations do not reach hazardous levels.

We have identified a natural clay mixture, designated CB, which has antibacterial activity against a broad spectrum of bacterial pathogens. Previously, we prepared aqueous clay mixture extracts (leachates) and demonstrated that the *in vitro* antibacterial activity of the natural clay sample is associated with the generation of low pH environment and the chemical desorption of ions from the surface of the clay particles [10]. However, as described in this report, we have collected four samples from the same source and demonstrated that the *in vitro* antibacterial activity of these minerals depends on the chemical properties of the clay mixtures. This discrepancy in antibacterial activity must be controlled if these clay mixtures or developed clay minerals are to be used therapeutically against topical bacterial infections. In order to define the properties that are associated with varied antibacterial efficacy, we characterized the mineralogical and geochemical composition of the four clay mineral samples. Furthermore, while metals ions have been shown to be toxic, the total ion concentration does not always correlate directly to toxicity. Rather, ion toxicity is directly linked to ion speciation changes influenced by the pH, redox state, ion solubility, osmotic strength, and temperature during experimental conditions [22]. We evaluated the interplay between total ion concentration, ion speciation, and leachate antibacterial activity by performing antibacterial susceptibility testing of leachates supplemented with additional ions and subjected to pH adjustments. Finally, we used Visual MINTEQ to model the speciation and solubility changes of ions present in the leachates to predict which species specifically contribute to the toxicity of the leachates.

## Materials and Methods

### Bacterial Strains and Growth Conditions

*Escherichia coli* ATCC 25922, obtained from the American Type Culture Collection, and methicillin-resistant *Staphylococcus aureus* (MRSA), obtained from Sonora Quest Laboratories (Tempe, AZ, USA), were used for all studies as previously described [7]. *E. coli* was grown on Luria-Bertani (LB) agar or in LB broth, and MRSA was grown on trypticase soy agar (TSA) or in trypticase soy broth (TSB). Both bacterial strains were grown at 37°C with gentle rotary mixing.

### Antibacterial Susceptibility Testing

*E. coli* and MRSA exponential phase cultures were prepared by diluting overnight cultures into fresh growth medium to a concentration of 10^7^ CFU/mL and continuing growth at 37°C with gentle rotary mixing until the cultures reached mid-logarithmic phase of growth. Bacterial cells were collected by centrifugation, washed once in 0.85% sterile saline, and suspended in the appropriate clay mixture leachate solution, clay mixture (at 1% or 10% w/v), or sterile UV-irradiated ultrapure H_2_O (dH_2_O) at an initial concentration of 10^7^ CFU/mL. Samples were incubated at 37°C with gentle rotary mixing for 24 h, and cell survival was determined by plating duplicate 10-fold serial dilutions for each sample at appropriate time points and enumerating colonies on plates after overnight incubation at 37°C. Due to sample processing, the indicated 0 h experimental exposure times were equivalent to approximately 3 min exposures.

### Clay Mixtures and Mineral Leachate Preparation

Clay mixtures were autoclaved for 1 h at 121°C before experimental use. A 10% CB suspension refers to 0.1 g of clay mixtures mixed in 1 mL sterile dH_2_O. CB-derived leachates (CB-L) were obtained by continuously stirring clay mixtures (1 g/20 mL) in sterile dH_2_O for 18–24 h. Subsequently, the hydrated clay mixture suspensions were centrifuged (31,000×g) for 3 h at 4°C to separate insoluble and soluble fractions. The aqueous supernatant (leachate) was collected and sterilized by passage through a 0.22 µm filter.

### X-ray Diffraction Sample Preparation and Analysis

Qualitative mineralogical profiles of CB07, CB08, CB09, and CB10 samples were analyzed as oriented mounts on glass slides from 2° to 35° two-theta with the standard treatments of K-saturation followed by heating to 350°C and 550°C, Mg-saturation, and Mg-saturation/glycerol solvation [16]. X-ray diffraction analyses were conducted at the University of Arizona Center for Environmental Physics and Mineralogy using a PANalytical X'Pert PRO-MPD X-ray diffraction system (PANalytical, Almelo, AA, The Netherlands) producing Cu–Kα radiation at an accelerating potential of 45 kV and current of 40 mA and fitted with a graphite monochromator and sealed Xenon detector.

Quantitative analyses of the four CB clay mixture samples were run as random powder mounts and measured from 5° to 65° two-theta. Samples were prepared by pulverizing in a McCrone micronizing mill (The McCrone Group, Westmont, IL) after the addition of a 20% (w/w) corundum internal standard. After measurement, diffractograms were imported into RockJock [17], a program for determining quantitative mineralogy from powder X-ray diffraction data, and analyzed with Reitveld refinement. Measurement conditions were as follows: 0.02° step size, 3 sec dwell time per step, spinning at 1 revolution per second, 1/4° divergent anti-scatter slit, 15 mm divergent mask, 1/4° incident anti-scatter slit, and 0.6 mm fixed receiving slit.

### ICP-MS/−OES Sample Preparation and Analysis

Measurements of the elemental concentrations in the leachate samples were determined using inductively coupled plasma mass spectrometry (ICP-MS) (ThermoQuest/Finnigan Element 2), and the clay mixture samples were analyzed by ICP-MS and inductively coupled plasma – optical emission spectrometry (ICP-OES) (Perkin Elmer Optima 3000). For clay mixture sample analysis, the procedures used were similar to those described by Janney *et al*. [18] and Solidum *et al*. [19] with some modifications. Prior to analysis, 0.025–0.030 g of sample powder was digested using ∼1 mL of a 2∶1 mixture of double-distilled and concentrated hydrofluoric (HF) and nitric (HNO_3_) acids and either placed in an ultrasonic bath for 60 minutes or heated at 60°C overnight. After the powder was fully dissolved, the sample-acid solution was evaporated to dryness over a hotplate at 60°C and under a heat lamp in a teflon evaporating unit. The digested residue was then treated twice with ∼0.5 mL of concentrated HNO_3_ and evaporated to dryness. For ICP-MS analyses, the digested sample powder was first diluted 100× (by weight) with a 2.5% HNO_3_ acid solution containing 1 ppb indium (In) as an internal standard, and then an aliquot of the digested sample solution was diluted ∼4000× with the 2.5% HNO_3_ solution containing indium. Calibration was achieved using one blank and a series of three synthetic standard solutions with elemental concentrations ranging from 0.05 to 10 ppb. For the ICP-OES analyses, an aliquot of the 100× diluted sample solution described above was diluted 2000× the sample weight using a 1% HNO_3_ acid solution. Instruments were calibrated with one blank solution and a series of four synthetic standard solutions with element concentrations ranging from 20 to 500 ppb in 1% HNO_3_ acid medium. The accuracy and precision of the analytical method were monitored by analysis of natural rock standards, AGV-1 and BHVO-1, prepared in the same manner as the unknown samples.

The leachate samples were analyzed on an ELAN DRC-II ICP-MS (Perkin Elmer, Shelton, CT). Calibration standards were prepared from multi-element stock solutions (except for Hg, which is a single element standard) purchased from AccuStandard (New Haven, CT). The stock solutions were diluted in 1% nitric acid to provide a working calibration curve of at least five measurements. Samples were also diluted with 1% nitric acid until their response was determined to be within the calibration range. Internal standards (Rh, In, and Ga) were added to both standards and samples prior to analysis.

### Leachate pH Adjustment and Ion Supplementation

For the ion supplementation experiments, CB09-L was prepared as described above and then subsequently supplemented with chloride salts of Fe, Co, Ni, Cu, and Zn to final ion concentrations equivalent to values present in natural CB07-L. Where indicated, the pH of the solutions was adjusted with 1 M HCl or 1 M NaOH to a pH of 3.4 or 5.0 to mimic the native pH values of CB07-L and CB09-L, respectively. Finally, the leachates were sterilized by passage through a 0.22 µm filter prior to further analyses.

### Oxidation-Reduction Potential Measurements

Half-cell oxidation-reduction potential (ORP) measurements were made using an Accumet Basic AB15 Plus pH meter equipped with a single junction Ag/AgCl reference electrode and a platinum ORP-indicating electrode. Final E_h_ values were calculated by adding the half-cell potential measurement for a Ag/AgCl electrode at 25°C (+199 mV) to the measured value. The electrode was calibrated with Zobell’s solution, and the ORP value was normalized to this value accordingly.

### Speciation Modeling Analysis of the Leachate Samples

Aqueous speciation and solubility of Fe, Co, Ni, Cu, and Zn were calculated using Visual MINTEQ (version 3.0) modeling software. The ICP-MS/OES-determined ion concentrations and pH of the CB leachates were used to calculate ion speciation and solubility. Experimentally determined E_h_ values used for each leachate (CB07-L, 567.1 mV; CB08-L, 514.2 mV; CB09-L, 524.0 mV; CB10-L, 567.5 mV) were included in the calculation of redox active ion pairs present in the leachates (Fe^2+^/Fe^3+^, Co^2+^/Co^3+^, and Cu^1+^/Cu^2+^).

### Statistical Analyses

Reduction in bacterial concentration was measured as the log_10_ decrease in bacterial viability following a 24 h exposure to the experimental conditions and tested using one-sample t-tests. Relative differences in antibacterial activity between two leachates were tested using two-sample t-tests with Satterthwaite approximation. Associations between ion species concentrations and antibacterial activity were assessed using linear regression of the log_10_ reduction in bacterial concentration against log_10_ ion species concentration. Multivariate regression models were constructed using stepwise regression and the Bayes Information Criterion. Analyses were conducted using R 2.15.3 statistical software.

## Results and Discussion

### CB Clay Mixtures and Leachates Exhibit Variable *in vitro* Antibacterial Activities

**Figure 1:**
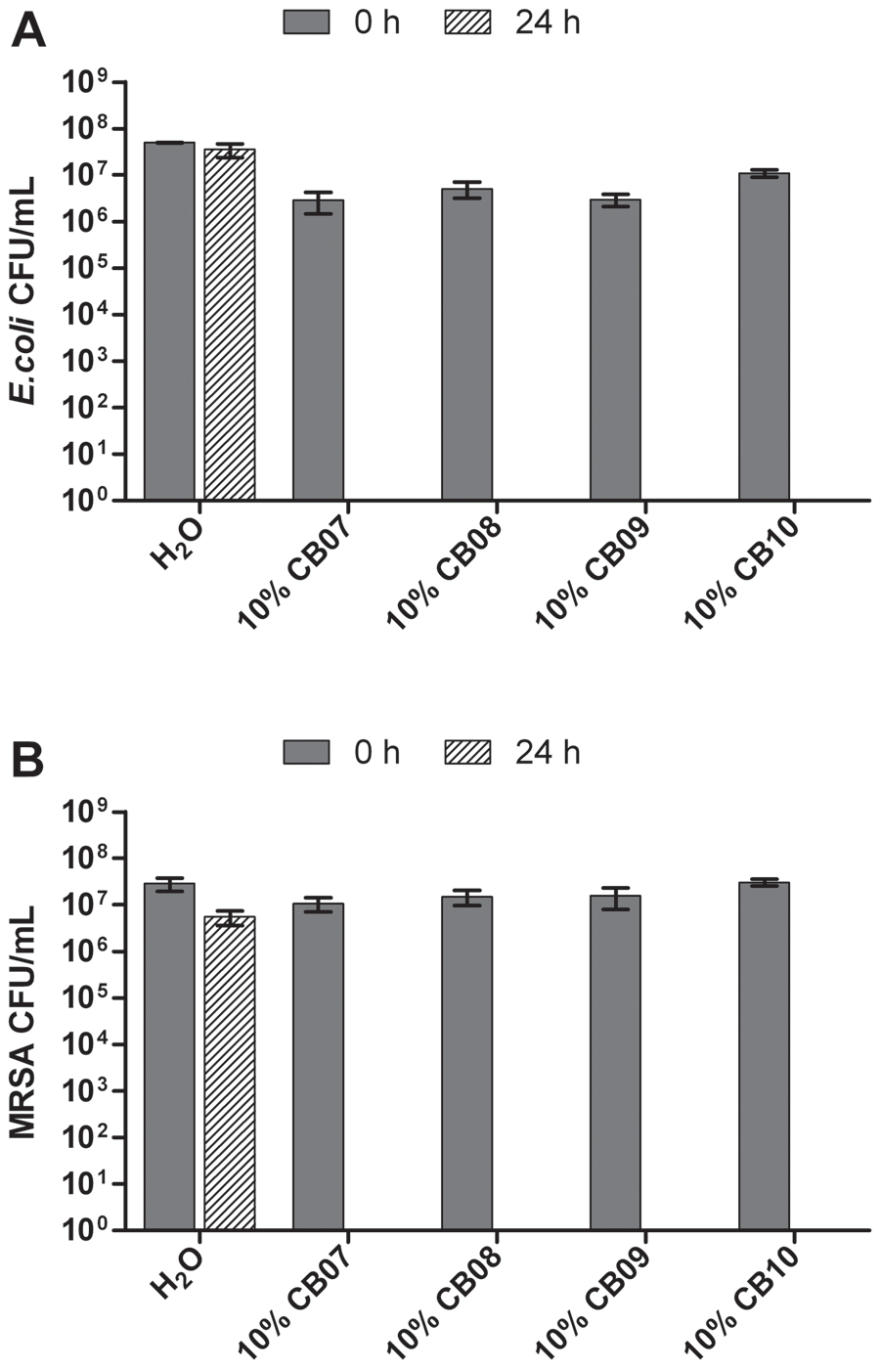
Viability of *E. coli* (A) and MRSA (B) following 24 h exposure to 10% suspensions of CB clay mixtures in dH_2_O. Error bars represent the standard error of the mean from at least three independent replicates.

**Figure 2:**
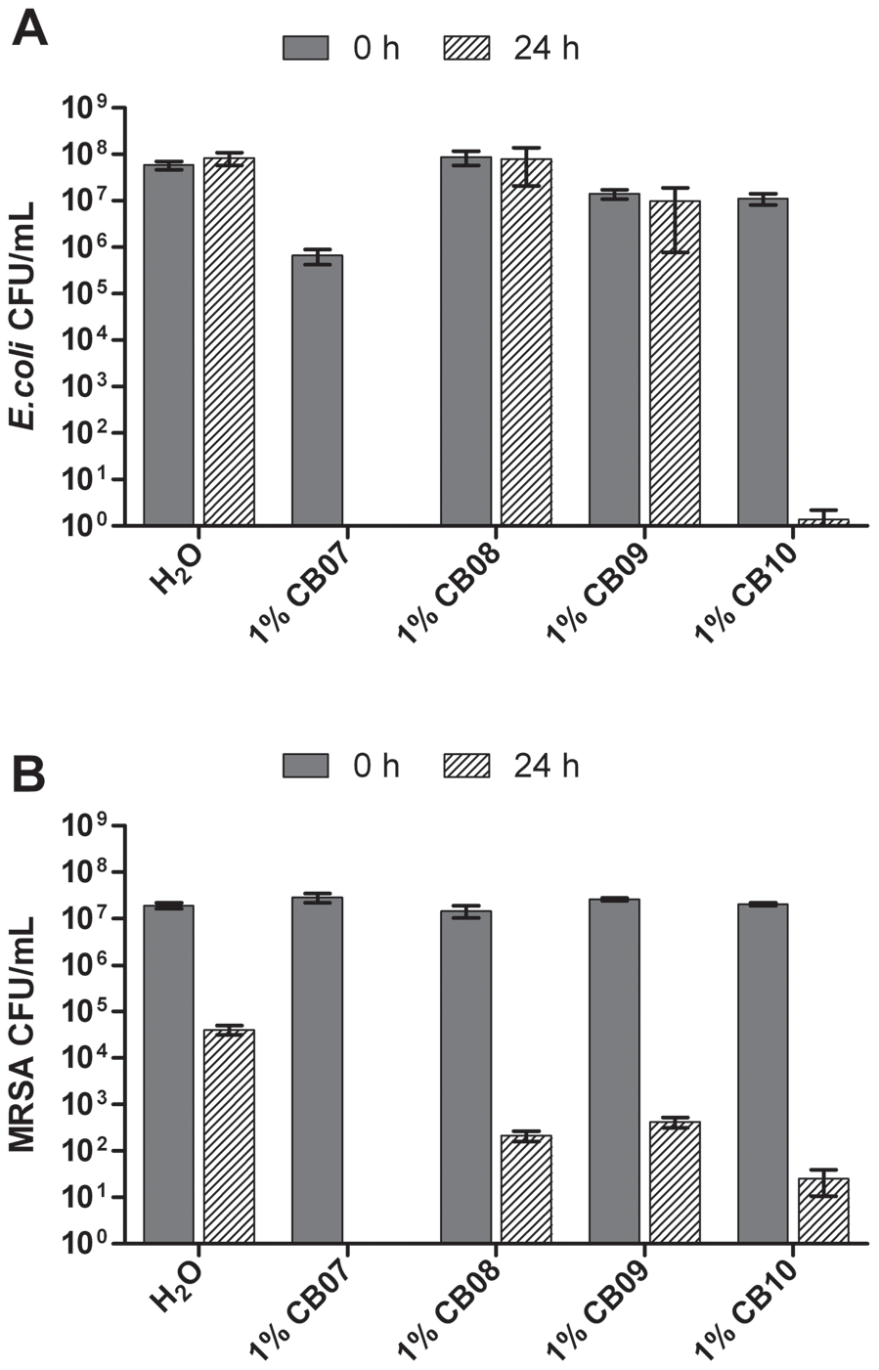
Viability of *E. coli* (A) and MRSA (B) following 24 h exposure to 1% suspensions of CB clay mixtures in dH_2_O. Error bars represent the standard error of the mean from at least three independent replicates.

**Figure 3:**
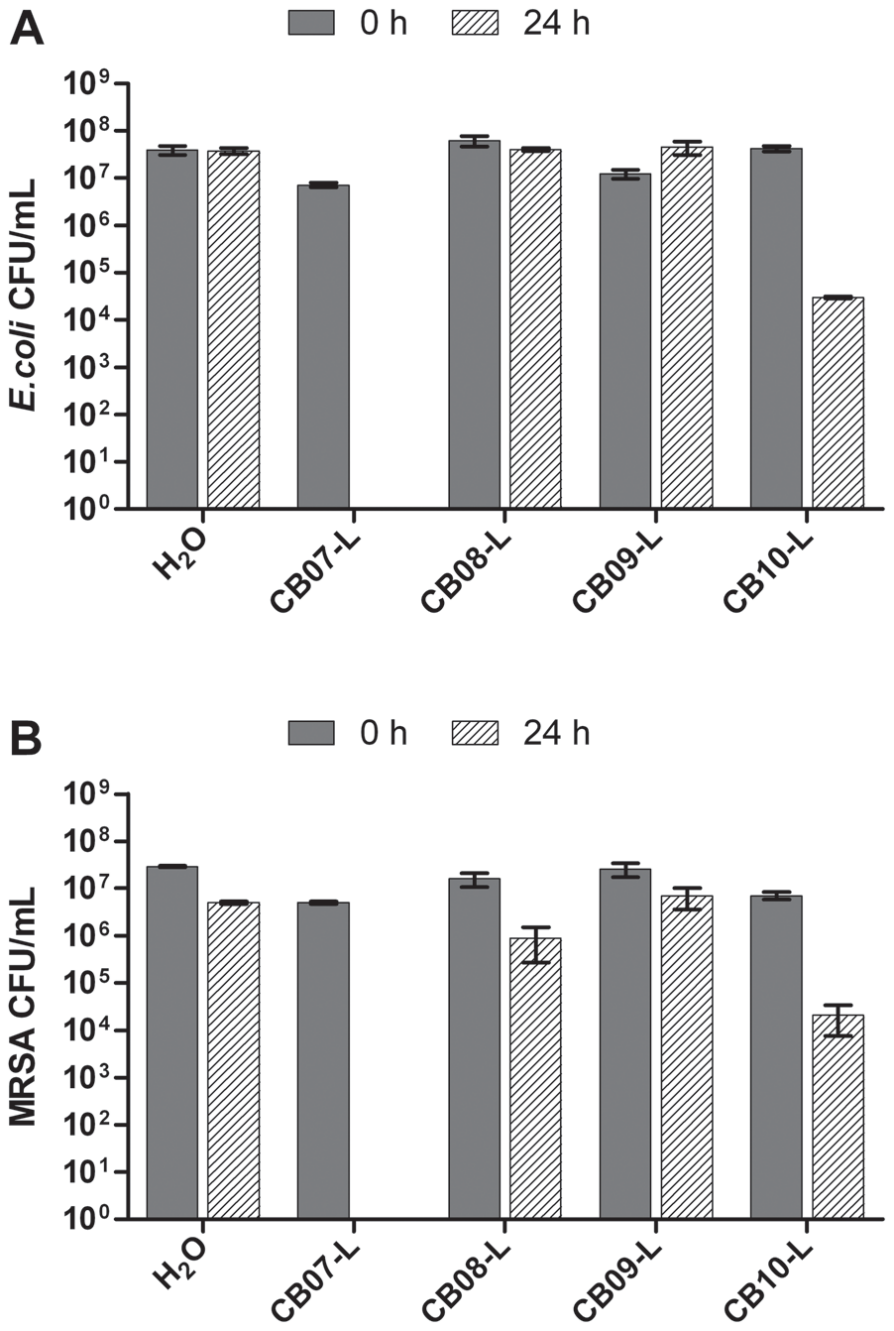
Viability of *E. coli* (A) and MRSA (B) following 24 h exposure to CB leachates. Error bars represent the standard error of the mean from at least three independent replicates.

*In vitro* antimicrobial susceptibility experiments with clay-water suspensions and leachates were performed to assess the effect of the CB clay mixtures on the growth of *E. coli* and MRSA. Ten percent suspensions of CB07, CB08, CB09, and CB10 completely killed *E. coli* and MRSA within 24 h (Figure 1). Exposure of *E. coli* and MRSA to 1% suspensions of CB07 and CB10 resulted in complete killing and nearly complete killing, respectively (Figure 2). However, when *E. coli* was exposed to 1% suspensions of CB08 and CB09, there was no decrease in viability (Figure 2A). Exposure of MRSA to 1% suspensions of CB08 and CB09 minerals resulted in bactericidal activity (4.8- and 4.7-log_10_ unit reductions, respectively), but did not completely kill the cells (Figure 2B). The effects of clay mixture leachate exposures to *E. coli* and MRSA are shown in Figure 3. CB07-L completely killed *E. coli* and MRSA, while CB10-L resulted in 3.1- and 2.5-log_10_ decreases in *E. coli* and MRSA viability, respectively (Figure 3). *E. coli* and MRSA viability was minimally decreased or affected following 24 h exposures to CB08-L and CB09-L, respectively (Figure 3). Overall, the CB07 clay mixture and CB07-derived leachate, followed by CB10 clay mixture suspensions and CB10-L, have the greatest antibacterial efficacy, compared to the other CB clay mixture and leachate samples.

### CB Clay Mixtures Are Mineralogically Similar

**Figure 4:**
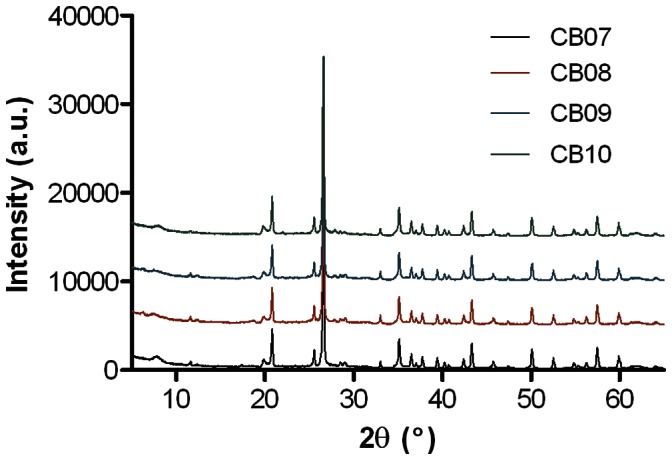
X-ray diffraction spectra of the CB clay mixtures.

X-ray diffraction (XRD) analyses of the CB minerals (Figure 4) revealed that the minerals are all composed of approximately 52% clays and 48% non-clay minerals (Table 1). The most abundant clay mineral in all of the samples was illite-smectite (36–37%), followed by montmorillonite (9.7–14.2%) and kaolinite (1.4–3.6%) (Table 1). The CB08 and CB09 samples also contained small amounts of chlorite (Table 1). The most abundant non-clay mineral present in the CB samples was quartz (34–37.3%), followed by pyrite (4–5.5%) and jarosite (2.4–4.7%). Overall, the XRD analyses of the CB minerals revealed minor mineralogical differences between the four samples.

**Table 1:** X-ray diffraction mineralogical analysis of the CB clay mixtures (% w/w).

Mineral		CB07	CB08	CB09	CB10
**NON-CLAYS**	Jarosite	4.7	4.5	3.7	2.4
	Quartz	37.3	34.4	34	36.6
	Albite	–	1.5	1.4	–
	Bytownite	–	–	–	2.8
	Pyrite	4	5.5	5.2	4.3
	Gypsum	2.5	2.9	3	1.7
	**Total non-clays**	**48.4**	**48.7**	**47.3**	**47.8**
**CLAYS**	Kaolinite	1.4	3.6	3.5	1.7
	Illite-Smectite	36	37	36.6	36.7
	Montmorillonite	14.2	9.7	11.7	13.8
	Chlorite	–	1	0.9	–
	**Total clays**	**51.6**	**51.3**	**52.7**	**52.2**

–, not detected.

### Compositions of Exchangeable Ions Released from CB Clay Mixtures and Present in CB Leachates Vary Across the Samples

Clay mineral surfaces are naturally negatively charged, thus allowing free exchange of positively charged species, such as metal cations. When in a hydrated suspension, these cations can be released from the surface of the minerals into the surrounding medium. Previously, we demonstrated that the physical interactions with the clay mixtures do not mediate antibacterial activity on their own, but rather, the freely exchangeable metal ions bound to the surface of the minerals are responsible for the antibacterial activity of CB07 clay mixtures [10]. ICP analyses of the four CB bulk clay mixture samples revealed that the ion compositions are similar, consistent with the mineralogical XRD data (Table 2). While the four CB samples were mineralogically similar, there were notable differences associated with the composition of ions bound to their surfaces (Table 3). For example, exchangeable Cu concentrations ranged from 0.04 to 3.18 µM, an approximate 80-fold difference in concentration across the four samples (Table 3). Likewise, exchangeable zinc concentrations ranged from 3.05 to 14.52 µM across the four leachate samples (Table 3). These ICP data revealed chemical variability across the samples, suggesting that the differences in antibacterial activity may be due to the variable concentrations of the exchangeable ions available in each of the clay mixture samples. However, while overall ion concentration is commonly assumed to be an accurate indicator of metal ion toxicity, it does not always accurately predict bactericidal activity [20]. Previously published experimental data and mathematical modeling [23] have demonstrated that Cu, Ni, and Zn toxicity increases with increasing pH [20]–[22]. The authors hypothesize that the pH-dependent changes in metal ion toxicity are due to increased metal-ligand interactions and increased concentrations of intracellular ions in the cases of Cu and Ni [20], [21] or changes in speciation over a defined pH range in the case of Zn [22]. With one exception, the concentrations of Fe, Co, Ni, and Zn were lower in CB10-L compared to the non-antibacterial CB08-L and CB09-L leachates, yet CB10-L antibacterial activity is maintained. The four CB leachates have a pH range of 3.4–5.0 (Table 3). Thus, while CB10-L has lower ion concentrations than the non-antibacterial leachates, the variable pH across the samples likely modulates speciation, solubility, and metal-cell interactions to affect antibacterial activity.

**Table 2:** ICP-OES and ICP-MS chemical analysis of the CB clay mixtures.

Element	CB07		CB08		CB09		CB10	
	ppm	mM	ppm	mM	ppm	mM	ppm	mM
Li	28.94	4.17	25.31	3.65	21.87	3.15	29.31	4.22
Be	1.18	0.13	0.97	0.11	0.91	0.10	0.74	0.08
Na	6514.32	283.36	8126.04	353.46	7399.97	321.88	4626.72	201.25
Mg	5736.95	236.04	10452.59	430.06	10452.59	430.06	3478.62	143.12
Al	81217.03	3010.27	87483.74	3242.54	80944.56	3000.17	67834.77	2514.26
Si	200088.70	7123.13	233132.40	8299.48	201952.30	7189.47	243049.16	8652.52
Si Wt %	42.80		49.87		43.20		51.99	
K	18544.45	474.31	16116.00	412.20	14623.66	374.03	10743.87	274.79
Ca	6909.29	172.39	11561.88	288.47	10349.06	258.21	4121.66	102.84
Ti	3104.93	64.87	3685.94	77.00	3685.94	77.00	2807.98	58.66
V	85.38	1.68	106.69	2.09	106.69	2.09	71.17	1.40
Cr	17.53	0.34	32.26	0.62	32.26	0.62	18.98	0.36
Mn	177.89	3.24	382.11	6.96	382.11	6.96	175.21	3.19
Fe	27580.00	493.87	34175.54	611.97	34175.54	611.97	24624.84	440.95
Co	47.97	0.81	42.63	0.72	42.63	0.72	2.73	0.046
Ni	23.15	0.39	35.35	0.60	35.35	0.60	10.48	0.18
Cu	18.88	0.30	30.76	0.48	27.74	0.44	21.51	0.34
Zn	41.60	0.64	54.38	0.83	54.38	0.83	43.78	0.67
Ga	19.90	0.29	19.59	0.28	18.25	0.26	18.54	0.27
As	1.17	0.010	1.41	0.013	1.38	0.012	0.45	0.0040
Rb	51.07	0.60	38.72	0.45	34.75	0.41	24.39	0.29
Sr	176.63	2.02	311.55	3.56	285.16	3.25	113.48	1.30
Y	7.91	0.089	8.25	0.093	7.41	0.083	4.79	0.054
Zr	40.78	0.45	32.97	0.36	32.87	0.36	53.67	0.59
Nb	4.93	0.053	4.85	0.052	4.69	0.050	4.59	0.049
Mo	6.94	0.072	4.75	0.050	4.83	0.050	4.78	0.050
Ag	–	–	–	–	–	–	13.32	0.12
Cd	105.07	0.93	96.83	0.86	101.71	0.90	85.15	0.76
Ba	399.57	2.91	630.27	4.59	1922.51	14.00	564.20	4.11
La	8.65	0.062	10.62	0.077	8.64	0.062	8.32	0.060
Ce	18.94	0.135	20.43	0.146	19.17	0.137	10.44	0.075
Pr	2.31	0.016	2.49	0.018	2.34	0.017	1.21	0.0086
Nd	9.48	0.066	10.53	0.073	9.80	0.068	4.62	0.032
Sm	1.93	0.013	2.13	0.014	1.97	0.013	0.93	0.0062
Eu	0.65	0.0043	1.28	0.0084	1.43	0.0094	–	–
Tb	0.25	0.0016	0.27	0.0017	0.25	0.0016	0.13	0.0008
Dy	1.37	0.009	1.46	0.0090	1.33	0.0082	0.72	0.0044
Ho	0.27	0.0016	0.29	0.0017	0.26	0.0016	0.14	0.0009
Er	0.73	0.0043	0.75	0.0045	0.69	0.0041	0.39	0.0024
Yb	0.79	0.0045	0.79	0.0046	0.70	0.0040	–	–
Lu	0.12	0.0007	0.12	0.0007	0.11	0.0006	–	–
Hf	1.04	0.0058	0.85	0.0047	0.84	0.0047	1.52	0.0085
Ta	0.33	0.0018	0.31	0.0017	0.30	0.0017	0.34	0.0019
Pb	13.80	0.067	18.14	0.088	18.01	0.087	7.97	0.039
Th	2.15	0.0093	2.06	0.0089	1.90	0.0082	1.42	0.0061
U	0.74	0.0031	0.79	0.0033	0.66	0.0028	0.74	0.0031

–, Indicates below the detection limit.

**Table 3:** ICP-MS chemical analysis of the clay mixture leachates.

Element	CB07-L (pH 3.4)		CB08-L (pH 4.7)		CB09-L (pH 5.0)		CB10-L (pH 4.2)	
	ppb	µM	ppb	µM	ppb	µM	ppb	µM
Li	52.86	7.62	12.61	1.82	8.03	1.16	49.66	7.15
Be	7.18	0.80	0.16	0.02	0.07	0.01	2.63	0.29
Na	3945.33	171.61	4490.55	195.33	4594.20	199.83	5774.46	251.17
Mg	18790.38	773.11	53742.79	2211.18	52845.95	2174.28	7633.77	314.08
Al	29127.64	1079.60	72.89	2.70	21.20	0.79	2297.69	85.16
Si	2697.00	96.03	2629.74	93.63	2873.29	102.31	5789.57	206.14
K	2490.00	63.69	4520.00	115.61	5930.00	151.67	1780.00	45.53
Ca	277000.0	6911.18	288000.0	7185.63	283000.0	7060.88	146000.0	3642.71
Ti	0.44	0.01	–	–	–	–	–	–
V	24.90	0.49	7.00	0.14	0.05	0.001	–	–
Cr	8.12	0.16	–	–	–	–	0.17	0.003
Mn	5455.65	99.31	7151.04	130.17	7052.63	128.37	3310.49	60.26
Fe	12837.74	229.88	5397.34	96.65	4867.39	87.16	471.63	8.45
Co	199.00	3.38	92.20	1.56	85.50	1.45	78.40	1.33
Ni	203.00	3.46	119.00	2.03	110.00	1.87	65.80	1.12
Cu	202.00	3.18	6.27	0.10	2.58	0.04	11.90	0.19
Zn	949.35	14.52	352.17	5.39	199.75	3.05	343.14	5.25
Ga	1.53	0.02	2.16	0.03	2.14	0.03	2.85	0.04
As	2.83	0.04	0.25	0.003	0.23	0.003	0.37	0.005
Rb	1.60	0.02	0.88	0.01	1.09	0.01	2.68	0.03
Sr	305.15	3.48	418.30	4.77	377.98	4.31	181.40	2.07
Y	158.00	1.78	19.20	0.22	11.30	0.13	21.40	0.24
Zr	0.09	0.001	–	–	–	–	–	–
Nb	–	–	–	–	–	–	–	–
Mo	0.23	0.002	–	–	–	–	–	–
Ag	–	–	–	–	–	–	–	–
Cd	1.39	0.01	1.89	0.02	1.68	0.01	0.53	0.00
Ba	8.30	0.06	12.40	0.09	12.80	0.09	16.70	0.12
La	56.70	0.41	9.89	0.07	6.73	0.05	17.00	0.12
Ce	183.00	1.31	24.90	0.18	15.30	0.11	41.10	0.29
Pr	25.00	0.18	3.68	0.03	2.15	0.02	5.71	0.04
Nd	35.10	0.24	6.49	0.04	2.84	0.02	5.34	0.04
Sm	33.50	0.22	3.96	0.03	1.99	0.01	5.90	0.04
Eu	13.10	0.09	1.57	0.01	0.79	0.01	2.30	0.02
Tb	5.58	0.04	0.65	0.004	0.32	0.002	0.90	0.01
Dy	28.10	0.17	3.31	0.02	1.57	0.01	4.52	0.03
Ho	4.75	0.03	0.59	0.004	0.29	0.002	0.77	0.005
Er	11.60	0.07	1.41	0.01	0.69	0.004	1.90	0.01
Yb	7.73	0.04	0.79	0.005	0.34	0.002	1.22	0.01
Lu	1.05	0.01	0.11	0.001	0.05	0.000	0.16	0.001
Hf	–	–	0.11	0.001	–	–	–	–
Ta	0.23	0.001	0.14	0.001	–	–	–	–
Pb	0.06	0.0003	–	–	–	–	–	–
Th	0.12	0.0005	0.002	0.0000076	0.0002	0.0000008	–	–
U	1.80	0.01	0.03	0.0001	0.01	0.0000295	0.26	0.001

–, Indicates below the detection limit.

### Ion Supplementation of Non-antibacterial CB09-L Confirms the Role of Exchangeable Ions in Clay Mixture Antibacterial Activity

**Figure 5:**
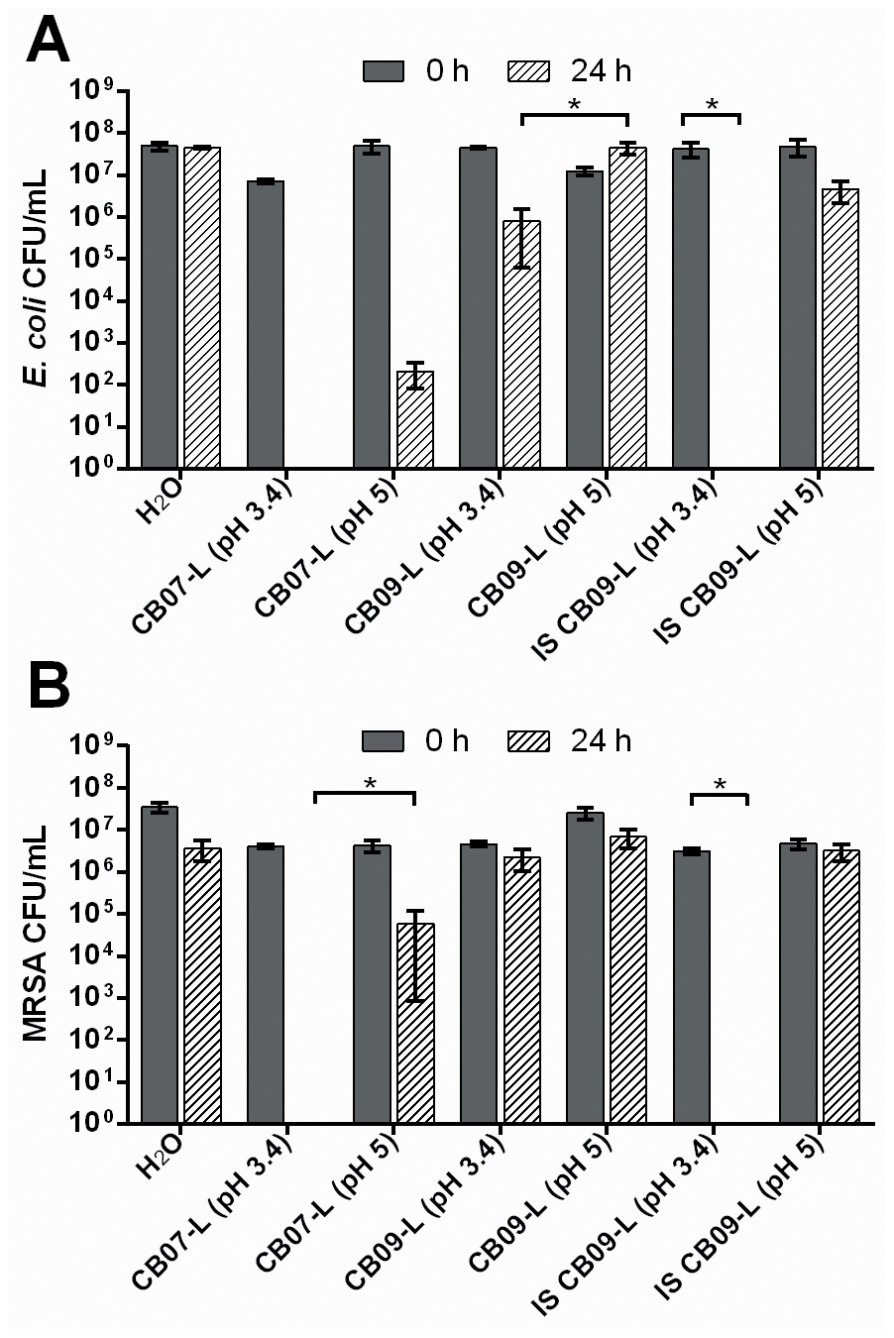
Viability of *E. coli* (A) and MRSA (B) following exposure to CB07-L, CB09-L, and CB09-L supplemented with Fe, Co, Ni, Cu, and Zn (IS CB09-L) each at a pH 3.4 or pH 5. Error bars represent the standard error of the mean from at least three independent replicates. * p<0.05.

To evaluate the influence of pH and ion speciation on the antibacterial activity, we modified the pH of CB07-L (antibacterial) and CB09-L (non-antibacterial) to that of the other respective leachate. The natural pH of CB07-L is 3.4 and was subsequently increased to a pH of 5 prior to antibacterial susceptibility testing. Likewise, the natural pH of CB09-L is 5.0 and was decreased to 3.4 for further testing. Figure 5 shows that increasing the pH of CB07-L rescued MRSA (4.7-log_10_ unit increase; p = 0.039) from killing. However, decreasing the pH of CB09-L to 3.4 only slightly increased the antibacterial activity of the leachate against *E. coli* (p = 0.018) and did not alter the antibacterial activity against MRSA.

Previously, we performed antibacterial susceptibility experiments whereby we supplemented the clay mixtures with metal chelators [10]. The addition of desferrioximine, which readily chelates Fe, Co, Cu, Ni, and Zn, rescued *E. coli* cells from clay-mediated killing [10]. Therefore, we hypothesized that these ions were major contributors to the antibacterial activity of natural clay mixtures. The ICP analyses revealed that the concentrations of these five ions are higher in the CB07 bactericidal leachate compared to the non-antibacterial counterparts, CB08-L and CB09-L. To confirm the role of these ions in the bactericidal activity of CB clay mixtures, we supplemented the non-antibacterial leachate, CB09-L, with Fe, Co, Ni, Cu, and Zn such that the final ion concentration of these elements and pH were equal to that of CB07-L (pH 3.4). Antibacterial susceptibility testing of the ion-supplemented CB09-L (IS CB09-L) resulted in complete killing of *E. coli* (p<0.001) and MRSA (p<0.001) following a 24 h exposure (Figure 5). When the IS CB09-L pH was subsequently increased from 3.4 to 5, it no longer killed *E. coli* or MRSA (Figure 5).

### Speciation Modeling Implicates Zn^2+^, Ni^2+^, Co^2+^, Cu^2+^, and Fe as Effectors of Antibacterial Activity

**Figure 6:**
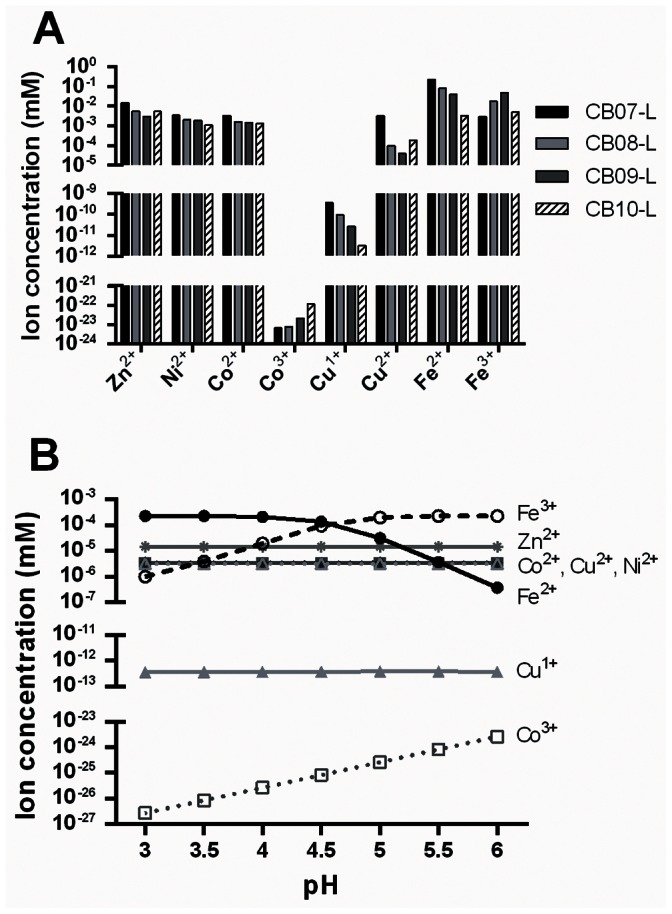
Concentrations and ionic species predicted to be present in the CB mineral leachates (A). Ion speciation (%) at respective ion concentrations present in CB07-L between pH 3–6 as predicted by Visual MINTEQ (B).

The concentration of total ions in the CB leachates did not directly correlate to antibacterial activity. Thus, we hypothesized that specific, toxic ionic species may be at higher concentrations in the antibacterial leachates, CB07-L and CB10-L, compared to the CB08-L and CB09-L non-antibacterial leachates. To test this hypothesis, we used Visual MINTEQ to model the ion speciation and solubility patterns in the CB leachates. Figure 6A shows the concentration and soluble ion species of Zn, Ni, Co, Cu, and Fe predicted to be present in the CB clay mixture leachates. CB07-L has the highest concentration of all measured species (with the exception of Fe^3+^ and Co^3+^), followed by CB08-L, CB09-L, and CB10-L (Figure 6A), which is consistent with the pattern of the total concentration of these ions in the leachates. Metals are more soluble in lower pH environments, thus the larger concentration of metals in CB07-L is likely due to the lower pH of this leachate. Of all the species tested, Cu^2+^ is the only species that is higher in both CB07-L and CB10-L than in CB08-L and CB09-L, making it a possible candidate for contributing to antibacterial activity. Since antibacterial activity is diminished with increasing pH, we modeled speciation changes that occur between pH 3 to 6 (Figure 6B). While the concentrations of Co^2+^, Cu^1+^, Cu^2+^, Ni^2+^, and Zn^2+^ did not change over this pH range, Fe^3+^ and Co^3+^ concentrations decreased, and the concentration of Fe^2+^ increased when pH decreased (Figure 6B). The increase of Fe^2+^ in a lower pH environment suggests its involvement in the antibacterial activity of the leachates.

Single variable regression analyses revealed that Zn^2+^, Ni^2+^, Co^2+^, and Cu^2+^ concentrations are positively associated with increased antibacterial activity against *E. coli*, while Zn^2+^, Co^2+^, and Cu^2+^ are positively associated with antibacterial activity against MRSA (p<0.01). These four species, Zn^2+^, Ni^2+^, Co^2+^, and Cu^2+^, were highly correlated (R^2^>0.93 for each pair of ion species), thus it remains unknown which one (or more) of these species is a key contributor to antibacterial activity. We constructed multivariate regression models to examine the combined role of different ion species and pH on antibacterial activity. The multivariate regression statistical models revealed that pH, Ni^2+^, Fe^2+^, and Fe^3+^ best explained antibacterial activity against *E. coli* (R^2^ = 0.904), while pH, Co^2+^, Co^3+^, Fe^2+^, and Fe^3+^ best explained antibacterial activity against MRSA (R^2^ = 0.786). We should note that a limitation of the speciation modeling and ICP analyses is that neither includes information about anions, such as sulfur anions, potentially present in the leachates. Sulfite, for example, increases in toxicity with decreasing pH, consistent with the toxicity pattern seen in the leachates [24]. The pyrite and jarosite minerals present in the CB clay mixtures are both sulfur composite minerals offering a potential source of sulfur to the clay mixture suspensions and leachate solutions. Thus, further analyses evaluating the presence and toxicity of anions must be performed to address anions as possible sources of toxicity.

### Conclusions

Overall, ICP analyses demonstrated that the concentrations of Fe, Co, Cu, Ni, and Zn vary greatly among the four CB mineral leachates. Non-antibacterial CB leachates supplemented with these additional ions and adjusted to a lower pH exhibited increased antibacterial activity. However, when the pH of the ion-supplemented leachate is increased, the solution loses antibacterial activity. Univariate regression analyses revealed that Zn^2+^, Ni^2+^, Co^2+^, and Cu^2+^ concentrations were positively correlated with antibacterial activity in *E. coli* and that Zn^2+^, Co^2+^, and Cu^2+^ concentrations were positively correlated with antibacterial activity in MRSA. While pH is an important factor in the speciation of metal ions, we demonstrated that killing activity is not solely attributed to pH. Together, these analyses further indicate that the concentration and speciation of exchangeable metal ions are responsible for antibacterial activity and that pH alone is not responsible for killing. Because of the natural variability across natural clay mixture samples and the correlated variability in antibacterial activity, efforts must be taken to standardize the composition of these minerals to ensure consistent antibacterial efficacy.
